# A continuous time meta-analysis of the relationship between conspiracy beliefs and individual preventive behavior during the COVID-19 pandemic

**DOI:** 10.1038/s41598-022-15769-4

**Published:** 2022-07-07

**Authors:** Lukasz Stasielowicz

**Affiliations:** Institute of Psychology, Osnabrück University, Seminarstraße 20, 49074 Osnabrück, Germany

**Keywords:** Psychology, Health care

## Abstract

In several longitudinal studies, reduced willingness to show COVID-19-related preventive behavior (e.g., wearing masks, social distancing) has been partially attributed to misinformation and conspiracy beliefs. However, there is considerable uncertainty with respect to the strength of the relationship and whether the negative relationship exists in both directions (reciprocal effects). One explanation of the heterogeneity pertains to the fact that the time interval between consecutive measurement occasions varies (e.g., 1 month, 3 months) both between and within studies. Therefore, a continuous time meta-analysis based on longitudinal studies was conducted. This approach enables one to examine how the strength of the relationship between conspiracy beliefs and COVID-19 preventive behavior depends on the time interval. In total, 1035 correlations were coded for 17 samples (*N* = 16,350). The results for both the full set of studies and a subset consisting of 13 studies corroborated the existence of reciprocal effects. Furthermore, there was some evidence of publication bias. The largest cross-lagged effects were observed between 3 and 6 months, which can inform decision-makers and researchers when carrying out interventions or designing studies examining the consequences of new conspiracy theories.

## Introduction

In January 2022, it was estimated that between 9 and 22 million more people died during the COVID-19 pandemic than one would expect had there been no pandemic^1^. Arguably, many of the deaths were preventable. During the unfolding of the pandemic, multiple preventive behaviors were proposed to reduce the spread of the virus, e.g., wearing masks and distancing. However, not all people were willing to adopt specific behaviors. Several contributing factors have been proposed, including boldness^2^. Paiva et al.^2^ showed that people who are able to remain calm in stressful situations and show a high degree of self-assurance are less likely to adhere to preventive measures. Furthermore, in a longitudinal study, people with dark personalities (e.g., manipulative and impulsive people) were more likely to report barriers to preventive behaviors such as lack of time or pressure from friends and family, which in turn was linked to lower adherence to preventive regulations^3^. The low willingness to engage in preventive behaviors was also partially attributed to misinformation and conspiracy beliefs^4,5^. This should not be surprising because even long before the COVID-19 pandemic, researchers have acknowledged that conspiracy beliefs can lead to indifference^6^. Such indifference could be a result of seeing any efforts as futile due to the existence of powerful conspiratorial forces. Alternatively, officially recommended efforts could be seen as a part of a conspiracy, and one should avoid following them. Irrespective of the specific motives (i.e., resignation or avoiding conspiratorial forces), in the context of the COVID-19 pandemic, indifference could translate into reduced support or adherence to preventive measures such as wearing masks or social distancing. The current meta-analysis examines the relationship between conspiracy beliefs and preventive behaviors using data from longitudinal studies conducted during the pandemic. In addition, the present study offers a new perspective by demonstrating the relevance of the duration of time intervals between measurement occasions. A summary of the previous research and the rationale for the present meta-analysis are provided in the following paragraphs.

During the pandemic, many conspiracy theories emerged that linked specific people (e.g., Bill Gates) or events (e.g., the rollout of 5G technology) to the pandemic to question its existence or to explain the supposed origins of the pandemic^7^. One meta-analysis confirmed the relevance of such specific conspiracy beliefs and general conspiracy mentality to COVID-19-related preventive behaviors^8^. The researchers identified a small negative relationship between conspiracy beliefs and preventive behaviors (attitude, intention, or actual behavior). To increase the plausibility of causal claims, the researchers additionally conducted a separate analysis based solely on longitudinal studies. Interestingly, they found evidence of reciprocal effects. Not only were earlier conspiracy beliefs negatively related to later preventive behaviors, but earlier preventive behaviors were also negatively related to later conspiracy beliefs, which confirms the relevance of conspiracy beliefs.

While the results of the mentioned meta-analysis are both important and useful, as they demonstrate the existence of the relationship and are consistent with causal explanations, there are some aspects that need to be considered when interpreting the results. First, the longitudinal part of the meta-analysis conducted by Bierwiaczonek et al.^8^ was based only on eight longitudinal studies, as more data were not available at that time. However, in the following months, several other studies emerged. Second, the longitudinal analysis was restricted to two measurement occasions when examining the cross-lagged effects. However, studies utilizing several waves are available, including six waves from the C19PRC project^7^, which could help identify more complex patterns. Third, the mentioned meta-analysis was based on the standard static (discrete) time view (T1, T2, etc.). The discrete time perspective is based on a less realistic assumption than the continuous time perspective^9,10^. Specifically, the continuous time perspective has the advantage that it accounts for the fact that the relationship between variables (e.g., conspiracy beliefs and COVID-19-related preventive behaviors) depends on the length of the time lag (e.g., 4 weeks vs 16 weeks). As different time lags are adopted both within and between studies, accounting for such differences can lead to important insights. In particular, a continuous time meta-analysis can be used to estimate the optimal time length between measurement occasions, which can help researchers and practitioners when planning new studies or interventions. It is important because time lags that are too short or too long can preclude identifying effects and lead to false conclusions. This has already been acknowledged by some researchers in this research field^5^, who emphasized that short intervals (for example, 1 or 2 weeks) correspond to small effects. However, what counts as (too) short intervals is unclear at the present stage. The current study aims to close this research gap.

The present meta-analysis addresses all the issues and research gaps described in the previous paragraph in that it includes more studies, considers all available measurement occasions, and offers a complementary view of the relationship between conspiracy beliefs and COVID-19-related conspiracy behaviors by adopting a continuous time perspective and accounting for different time lags between and within studies. Furthermore, a continuous time meta-analysis has important implications for future empirical studies because it offers specific suggestions with respect to the optimal time interval between measurement occasions^11^.

Another reason for examining the role of the time lag is that the length of the time interval could be an explanation for inconsistent findings across studies. To illustrate, in one study^5^, evidence was found through a cross-lagged panel model that earlier conspiracy beliefs are related to later preventive behaviors (social distancing) but not vice versa. A similar pattern was found in a more recent study^12^. In contrast, one group of researchers reported reciprocal effects between conspiracy beliefs and preventive behaviors^13^. In another study, reciprocal effects were found only when examining general conspiracy mentality but not COVID-19-related conspiracy beliefs^14^. Thus, there is mixed evidence for reciprocal effects, and the time gap between measurement occasions is one explanation worth examining^5^.

Nevertheless, it is also worth asking whether reciprocal effects are plausible at all. The notion that conspiracy beliefs reduce preventive behavior is rather intuitive. Conspiracy theories are thought to lead to indifference^6^, and it has been speculated that they induce feelings of powerlessness, which could decrease engagement in preventive behavior^5^. However, Bierwiaczonek and colleagues note that in the context of a pandemic, it is possible that people believing in conspiracy theories do not think that the pandemic is real and do not see a need to show preventive behavior. Is the notion that preventive behaviors reduce conspiracy beliefs equally plausible? One research group^14^ offers some arguments for this direction of effect. Individuals might increase preventive behaviors for several reasons. Some people could eventually recognize that government and health institutions are indeed trying to contain a pandemic, and this increased trust could lead to increased preventive behaviors. It is also possible that under social pressure to follow official guidelines, people could change their behavior. Increased preventive behavior could then reduce conspiracy thinking by means of rationalization. People could change their behavior and adjust their conspiracy beliefs later to maintain consistency between their behavior and beliefs. Thus, reciprocal effects are plausible in this context, and heterogeneous findings could indeed be due to varying time lags. Therefore, the magnitude of both types of effects and the relevance of time intervals will be examined in the present meta-analysis. The meta-analytic steps, including continuous time modeling, are described in the following section.

## Methods

### Inclusion criteria

The studies needed to fulfil the following criteria to be included in the present meta-analysis: (1) longitudinal study, (2) conspiracy beliefs (COVID-19-related conspiracy beliefs or general conspiracy mentality) and COVID-19-related preventive behaviors (wearing masks, social distancing, hygiene, vaccination, or measures assessing different types of behavior simultaneously) were assessed, and for at least one of the two constructs two measurement occasions were available, and (3) participants experienced no intervention aimed at changing conspiracy beliefs or preventive behaviors during the study. There were no language restrictions in the current meta-analysis, but all potentially relevant articles were written in English.

### Literature search

Considering that the first SARS-CoV-2 infections were reported in 2019 (hence COVID-19), the literature search was restricted to studies published since 2019. To maximize the odds of identifying relevant articles, three databases were chosen (one very broad database and two collections of social science research). In PsycINFO and Web of Science, the following search string was used: "longitudinal" AND "conspir*" AND "covid" AND ("vacc*" OR "distanc*" OR "isolat*" OR "mask*" OR "hygien*" OR "adheren*"). In Google Scholar, multiple strings were used, e.g., "longitudinal study" "conspiracy" "covid" "vaccine". The full list of search strings and the number of hits is available at https://osf.io/k4ba9/.

**Figure 1:**
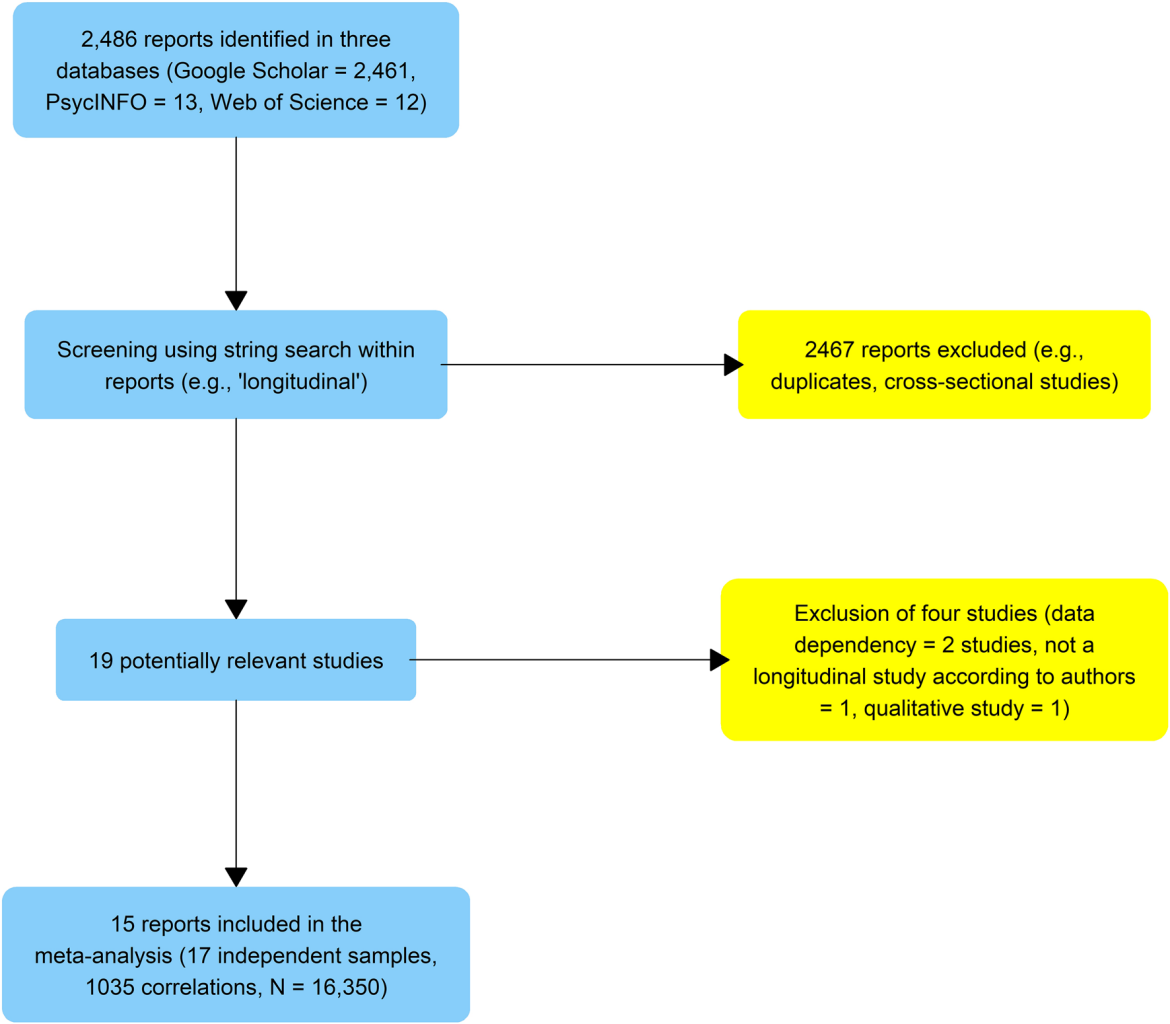
Flow chart summarizing the literature search.

The literature search was conducted over 9 days in November 2021, and the whole process is summarized in Fig. 1. In total, 2486 entries were screened (Google Scholar—2461; PsycINFO—13; Web of Science—12). Whenever available, the full text of reports was analyzed to decide whether the inclusion criteria were met. As raw correlation matrices or access to data sets are required for a continuous time modeling meta-analysis, some authors were contacted to provide the necessary information (see supplementary materials for a summary of contact attempts). Overall, 17 independent samples from 15 studies were identified.

### Coding

Each sample was coded twice (LS and MW). In addition to general characteristics (publication year, first author, country, etc.), information required for continuous time modeling was extracted. Specifically, time lags between measurement occasions (in months), sample sizes, and correlation matrices involving the relationships between conspiracy beliefs (CB) and preventive behaviors (PB) across all measurement occasions were coded. Both general conspiracy beliefs and COVID-19-related conspiracy beliefs were considered in the present continuous time meta-analysis, as it has been reported that general conspiracy beliefs are also relevant to COVID-19-related preventive behavior^8,14^.

Multiple effect sizes were available for many of the studies included in the current meta-analysis; for example, some researchers assessed both general and COVID-19-related conspiracy beliefs^12^. There were also cases where COVID-19 prevention was measured differently, e.g., social distancing and vaccination^7^. If multiple effect sizes were reported, a mean correlation was estimated for dependent effects. Extracted data are available at https://osf.io/k4ba9/.

### Statistical procedures

To examine the relevance of time lag length, the correlation coefficients from individual studies based on conspiracy beliefs and/or COVID-19-related preventive behaviors across all available measurement occasions were aggregated by means of continuous time modeling meta-analysis (CoTiMA). The respective analyses were conducted using the R package *CoTiMA*. The main difference between CoTiMA and standard meta-analytic approaches is the fact that, by using differential equations, time is regarded as a continuous rather than discrete variable^9,15^. This approach avoids the loss of information that is introduced by the arbitrary classification of time periods such as short, medium, and long. This is important because the strength of the relationship between variables often varies across time. In certain contexts, it is even possible that the sign of the relationship changes with time. To illustrate, antidepressants could have positive short-term effects and negative long-term effects^16^. Furthermore, CoTiMA allows one to include all available waves instead of excluding some waves due to data dependency. It has also been shown that CoTiMA estimates are less biased than estimates based on other approaches aimed at aggregating cross-lagged effects^9,10^.

Based on the correlations from individual studies, CoTiMA yields drift coefficients^9,10^, which consist of auto effects and cross effects. Auto effects refer to the relationship between different measurement occasions of the same variable (for example, conspiracy beliefs across time). In contrast, cross effects describe the relationship between different variables (e.g., conspiracy beliefs and prevention behavior) measured on different occasions. Thus, cross effects are of interest if one wants to examine reciprocal effects. The terms *auto effects* and *cross effects* rather than *autoregressive effects* and *cross-lagged effects* are used in continuous time modeling to avoid confusion with discrete time modeling in typical cross-lagged models^9,15^. In the present meta-analysis, the drift matrix consists of four coefficients: (1) auto effect for conspiracy beliefs; (2) auto effect for preventive behavior; (3) cross effect of conspiracy beliefs on preventive behavior; and (4) cross effect of preventive behavior on conspiracy beliefs.

To obtain the meta-analytic estimates of drift coefficients, first, a continuous time structural equation model is fitted separately to every sample. In the next step, the individual coefficients are aggregated across studies^9^. In contrast to a standard meta-analysis, the CoTiMA estimates of effects are not a simple weighted average of available studies, however. The likelihood values of individual studies are considered simultaneously to find the best set of auto effects and cross effects. For the purpose of facilitating interpretation of the results, the meta-analytic drift coefficients can be transformed into autoregressive and cross-lagged effects for arbitrarily chosen interval lengths.One could obtain effects for time lags of 3 months, 8 months or other intervals. This enables one to demonstrate how the effects depend on the choice of time lags.

Following the main analyses, publication bias analyses were conducted. In addition to coding the type of report (e.g., journal article, working paper), the relationship between individual effects and precision of the estimates was assessed by inspecting the funnel plots, systematically examining the funnel plot asymmetry with Egger’s intercept test^17^, and applying PET-PEESE (precision-effect test and precision-effect estimate with standard errors) to the available data^18^. The results of the current meta-analysis are summarized in the next section. First, general information about the included samples is provided, followed by a summary of the meta-analytic results and publication bias analyses.

## Results

### Descriptive statistics

In total, 1035 correlations were extracted for the 17 available samples (*N* = 16,350). The maximal sample size recorded for each sample varied between 110 and 5364 (Mdn = 546). With the exception of one international sample^4^, all studies were conducted in Western countries. Specifically, there were seven samples from the USA, three samples from the UK, two samples each from Germany and Poland, and one sample each from Australia and Ireland. In most studies, the mean age of the sample reflected the median age in the respective country quite well (Mdn = 40, Min = 24, Max = 48) when comparing the sample values to official records^19^. With respect to gender, in some samples, men were underrepresented or overrepresented, but in general, the percentage of men in the sample reflected the distribution in the particular country (Mdn = 49%, Min = 29%, Max = 57%).

The authors of the included studies used between one and five^14^ different measures of conspiracy beliefs per sample (*M* = 1.82). In 13 out of 17 samples, various COVID-19 conspiracy beliefs were assessed (e.g., the origin of the virus and vaccine). There were also two scales devoted solely to conspiracy beliefs with regard to vaccines against COVID-19^7,20^ and one scale devoted to supposed conspiracies of scientists and health institutions^7^. In seven studies, general conspiracy mentality was assessed, and in six of them, the Conspiracy Mentality Questionnaire was used^21^. The full table documenting instruments used in primary studies is provided in the supplementary materials.

With regard to measures of preventive behavior, the number of instruments varied between one and eight^7^ across primary studies (*M* = 1.94). In 11 of the 17 samples, scales were used, which assessed various preventive behaviors simultaneously. However, there were also studies in which specific behaviors were assessed separately. There were nine such studies assessing vaccination, four studies measuring social distancing, two studies assessing mask wearing, and two studies measuring hygiene. In general, researchers used scales that were developed specifically for the respective study. The full list is provided in the supplementary materials.

The number of waves per sample varied between two and six (Mdn = 2, *M* = 2.94). The shortest time interval between consecutive measurement waves was approximately 1 week^5,22,23^, and the longest time interval was more than 5 months^14^. The overall duration of the included studies varied between approximately 2 weeks^22,24^ and approximately 18 months^7^. However, the average duration was approximately 3 to 4 months (Mdn = 3.38, *M* = 4.34).

### Main results

**Figure 2:**
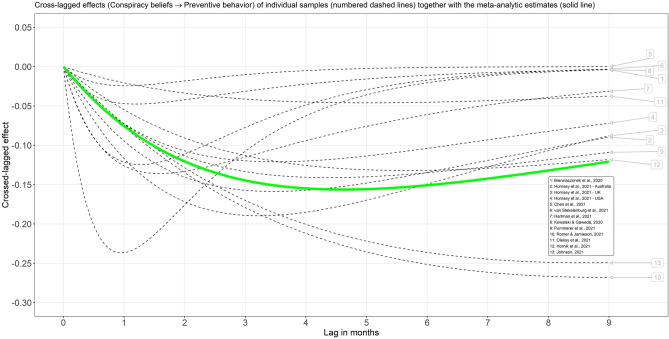
Model-implied cross-lagged effects (conspiracy beliefs → preventive behavior) for a range of time lags in months. On average, data for approximately 3 to 4 months were available. Thus, parts of the individual trajectories are simple extrapolations. For short studies, such as 1 and 8, the cross effects at large time lags cannot be reliably estimated. Hence, the largest effects in short studies are shifted to shorter time lags than the meta-analytic curve.

**Figure 3:**
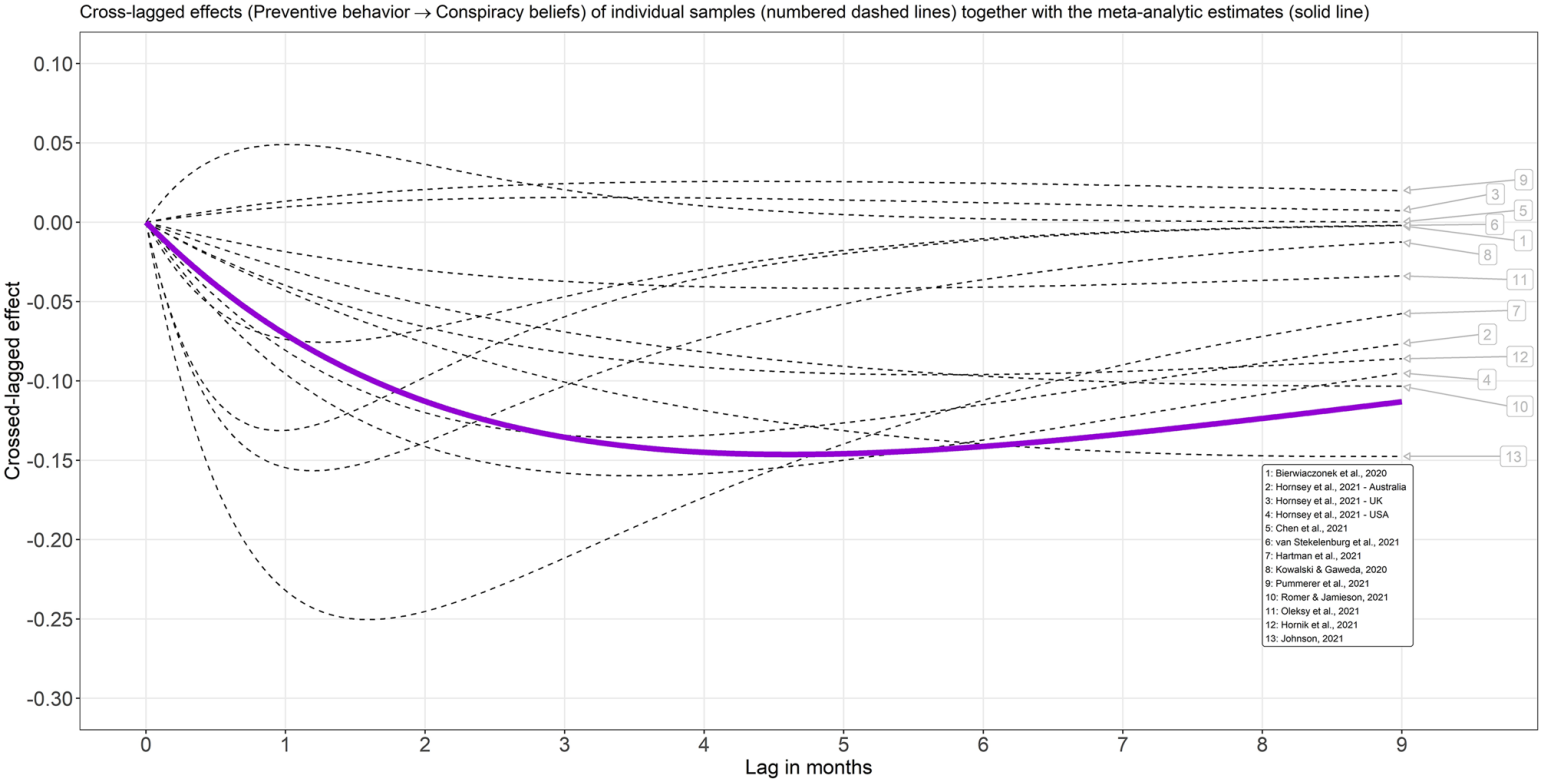
Model-implied cross-lagged effects (preventive behavior → conspiracy beliefs) for a range of time lags in months. On average, data for approximately 3 to 4 months were available. Thus, parts of the individual trajectories are simple extrapolations. For short studies, such as 1 and 8, the cross effects at large time lags cannot be reliably estimated. Hence, the largest effects in short studies are shifted to shorter time lags than the meta-analytic curve.

Initially, a CoTiMA based on all 17 samples was carried out. However, it is important to note that in two of the included studies, preventive behavior was assessed only once^4,25^, and in two further studies^26,27^, conspiracy beliefs were assessed just once, which means that not all auto and cross effects could be estimated reliably for these samples. To reduce the bias of the meta-analytic estimates, the all-invariant fixed effect model was used in the initial analysis, which assumes that not only drift effects (auto effects, cross effects) but also correlations at the first measurement occasion and diffusions (error variances and covariances) are equal (invariant) across studies. Nevertheless, the estimates for some of the primary studies mentioned above were visibly elevated, which could upwardly bias the final meta-analytic cross effects and auto effects: ${b}_{CB\to PB}$ = − 0.33, 95% CI [− 0.36, − 0.31], ${b}_{PB\to CB}$ = − 0.28, 95% CI [− 0.31, − 0.26]. Therefore, an additional CoTiMA was conducted. It included 13 samples for which at least two waves of measurements of both conspiracy beliefs and preventive behavior were available. It enabled the use of a less restrictive and more realistic model, in which only drift coefficients are assumed to be invariant. The meta-analytic estimates of all drift coefficients based on this modified model are reported in Table 1. In addition, the resulting model-implied cross-lagged effects are depicted in Figs. 2 and 3.

**Table 1:** Descriptive statistics and results of continuous time meta-analysis (drift coefficients) for the relationship between conspiracy beliefs and COVID-19-related preventive behavior in studies with at least two waves for both constructs.

	Value	95% CI
*k*	13	
*N*	12,696	
Optimal time lag	5	
**Cross effects**		
CB → PB	− 0.09	[− 0.11, − 0.08]
PB → CB	− 0.09	[− 0.10, − 0.08]
**Auto effects**		
CB	− 0.23	[− 0.24, − 0.21]
PB	− 0.23	[− 0.24, − 0.22]

*k* = Number of independent samples; *N* = Sample size; Optimal time lag = Time lag (in months) with largest meta-analytic cross-lagged effects (see also Figs. 2 and 3); CB = Conspiracy beliefs; PB = COVID-19-related preventive behavior (e.g., social distancing, wearing masks); For cross effects based on all 17 samples, see the main text (full output is available in supplementary materials).

While the magnitude of the cross effects was smaller than in the initial model, both cross effects differed from zero (${b}_{CB\to PB}$ = − 0.09, 95% CI [− 0.11, − 0.08]; (${b}_{PB\to CB}$ = − 0.09, 95% CI [− 0.10, − 0.08]). Thus, earlier conspiracy beliefs were related to later COVID-19 preventive behavior, and earlier COVID-19 preventive behavior was related to later conspiracy beliefs. To facilitate the interpretation of the coefficients, it is possible to transform drift coefficients into cross-lagged effects (or autoregressive effects) by simply multiplying the matrix of drift coefficients by the desired time lag (e.g., 5 months) and exponentiating the resulting matrix^9,10^:

$${\mathcal{e}}^{(D*\Delta t)},$$

where D is the drift matrix and Δt is the desired time lag. To illustrate, for a time lag of 5 months, the equation yields the following cross-lagged effects and autoregressive effects:

$${\mathcal{e}}^{\left(\left[\begin{array}{cc}-0.228& -0.089\\ -0.095& -0.232\end{array}\right]*5\right)}=\left[\begin{array}{cc}0.354& -0.146\\ -0.156& 0.347\end{array}\right]$$

The positive autoregressive effects for a time lag of 5 months simply mean that earlier conspiracy beliefs are related to later conspiracy beliefs (${b}_{CB}$ = 0.354) and earlier preventive behavior is related to later preventive behavior (${b}_{PB}$ = 0.347). The cross-lagged effect of conspiracy beliefs on preventive behavior (${b}_{CB\to PB}$ = − 0.156) means that people who have conspiracy beliefs greater by one unit than other people show less COVID-19-related preventive behavior by − 0.156. Analogously, ${b}_{PB\to CB}$ = − 0.146 means that people who show preventive behavior to a greater extent (by one unit) than other people believe less in conspiracy theories, and the difference is − 0.146. However, the magnitude of the discrete cross-lagged effect varies by the length of the time interval. Nevertheless, it is straightforward to insert time lag values other than 5 months into the formula to obtain cross-lagged effects for desired time intervals. For ease of interpretability, the magnitude of the meta-analytic cross-lagged effects is displayed in Figs. 2 and 3 for a range of different time lags. It appears that the largest cross-lagged effects can be expected when using a time lag of 5 months, but similar magnitudes can be expected between three and six months. Although trajectories of some primary studies appear to deviate from the meta-analytic estimates, it is largely due to study duration. After all, studies that ended after a few weeks cannot reliably estimate the effects for greater time lags. Therefore, the largest cross-lagged effects for relatively short studies^5,24^ were predicted for a time lag of approximately one month. In contrast, the largest effects for longer studies^20,28^ were predicted for greater time lags. Due to statistical artifacts (biased estimates from short studies), different trajectories should not be equated with true heterogeneity.

### Publication bias

Several analyses were carried out to examine publication bias. The results are summarized in Table 2, and the full output, including funnel plots, is available in the supplementary materials. PET-PEESE analyses yielded slightly smaller estimates of cross effects for the relationship between earlier conspiracy beliefs and later COVID-19-related preventive behavior (original: − 0.09, PET = − 0.04, PEESE = − 0.07, PET-PEESE = − 0.07). For the relationship between earlier preventive behavior and later conspiracy beliefs, the corrections were stronger (original: − 0.09, PET = − 0.02, PEESE = − 0.04, PET-PEESE = − 0.02). When using Egger’sintercept test for asymmetry in the distribution of effect sizes, asymmetry was identified only for the first cross effect, such that larger (more precise) studies were associated with smaller (more positive) cross effects. Finally, since almost all included studies were already published as journal articles, it was not possible to use publication status as a proxy variable for publication bias in the present meta-analysis. Overall, there was some evidence for publication bias, particularly for the cross effect for the relationship between earlier conspiracy beliefs and later COVID-19-related preventive behavior. The magnitude of the meta-analytic estimates from the main analysis appears to be biased upwardly. The true effects might be less negative.

**Table 2:** Publication bias analyses.

Cross effect	Original estimate	Corrected estimates			Egger’s intercept test			
		PET	PEESE	PET-PEESE	${b}_{0}$	${p}_{0}$	${b}_{Precision}$	${p}_{Precision}$
CB → PB	− 0.09	− 0.04	− 0.07	− 0.07	− 2.39	0.035	− 0.04	0.063
PB → CB	− 0.09	− 0.02	− 0.04	− 0.02	− 2.07	0.074	− 0.01	0.451

The original and corrected meta-analytic point estimates (PET-PEESE) are displayed together with the results of Egger’s intercept test.

*k* = 13; *N* = 12,696; PET = precision-effect test; PEESE = precision-effect estimate with standard errors; CB = Conspiracy beliefs; PB = COVID-19-related preventive behavior (e.g., social distancing, wearing masks); Full R output is available in supplementary materials.

## Discussion

The present continuous time meta-analysis builds on the findings of Bierwiaczonek et al.^8^, who examined the relationship between conspiracy beliefs and COVID-19-related preventive behavior (e.g., social distancing, wearing masks, vaccination). The current quantitative synthesis corroborates the main findings of the previous meta-analysis. It also offers additional insights by including more longitudinal studies and including multiple waves per study. Furthermore, the present meta-analysis closes a research gap by accounting for varying time intervals both between and within studies and demonstrating the relevance of the duration of time intervals. The main findings and their implications for future research as well for decision-makers are summarized in the following paragraphs.

Conspiracy beliefs can sometimes be regarded as a threat^5^, and the present results corroborate this in the context of a pandemic. Specifically, earlier conspiracy beliefs were negatively related to later COVID-19 preventive behavior, e.g., people believing in conspiracy theories are less willing to wear masks or engage in social distancing. This is consistent with the assertion that conspiracy beliefs can impede dealing with a pandemic, which could potentially overwhelm the health system. The current findings are also consistent with the assertion that the relationship between conspiracy beliefs and preventive behavior is reciprocal. Thus, an increase in preventive behavior corresponds to a decrease in conspiracy beliefs. However, the specific mechanism (e.g., social pressure, increasing trust in health institutions) could not be addressed in the current meta-analysis, and further studies are needed. Nonetheless, the current findings elucidate the relationship between conspiracy beliefs and preventive behavior, as previously mixed findings have been reported with respect to reciprocal effects^5,12–14^. It could also be confirmed^5^ in the present study that the varying length of the time interval explains the superficially inconsistent findings across studies.

Examining the relevance of the time interval is one of the main contributions of the current study. The results are relevant to both decision-makers such as politicians and researchers, as they show that the strength of the relationship between conspiracy beliefs and preventive behavior depends on the choice of the time interval. The strongest relationships are expected between 3 and 6 months. This finding can inform decisions in the future, as it offers an estimate with respect to how much time practitioners and researchers have until the maximal negative effects of conspiracy beliefs are reached. There may be a window of opportunity in which measures can be deployed to largely contain the potential harm of uncontrollable dissemination of conspiracy theories. To illustrate, one could approach well-known people to communicate via social media their experiences with vaccines to reduce vaccine hesitancy^26^ or address safety concerns such as the supposed impact of vaccines on fertility^27^. Furthermore, as certain events give rise to new conspiracy theories (e.g., 9/11, rollout of 5G technology), further conspiracy theories can be expected to emerge in the future, and researchers can use the present estimates of the optimal time lag between measurement occasions when planning studies examining the impact of new conspiracy theories or designing interventions aimed at reducing conspiracy beliefs. However, it is important to consider both potential benefits (e.g., mental health, reduced long-term costs for health systems) and costs (e.g., staff, infrastructure, increased short-term expenditures) when applying interventions at a large scale. To illustrate, in the past, one research team conducted a thorough cost–benefit analysis of psychological therapy in the UK^29^. The authors estimated the potential costs of the intervention and looked at several potential benefits, including increased employability and reduced costs for a welfare state. The field of conspiracy research could also benefit from such analyses. Presumably, such cost–benefit analyses would need to account for specific differences between countries, including laws or infrastructure, or be restricted to a specific region.

## Limitations and future research directions

Although the present continuous time meta-analysis has some advantages over the previous meta-analysis based on the standard approach^8^, it also has some limitations. According to the results of publication bias analyses, the real magnitude of cross effects might be smaller. However, it should be noted that publication bias analyses for the final CoTiMA model were based on relatively few studies. Thus, there is some uncertainty with regard to the existence of publication bias in this research field, and the reported publication bias findings need to be interpreted with caution.

Importantly, no samples from Asia, Africa, or South America were available when conducting the present meta-analysis. However, even within Europe, there is some heterogeneity with respect to preventive behaviors such as vaccination^27^. Thus, the present meta-analysis is not restricted to a very homogenous sample of countries. In fact, there was heterogeneity both between and within studies, including different country-level rules and different operationalizations of preventive behavior (attitude, intention, actual behavior) and conspiracy beliefs (COVID-19-related conspiracy beliefs vs. general conspiracy beliefs). This means that the meta-analytic estimates of cross effects are not necessarily generalizable to certain contexts. It is reasonable to assume that the plausibility of COVID-19-related conspiracy beliefs varies across the pandemic^7,14^. Similarly, preventive behavior may change. To illustrate, during the pandemic, vaccine hesitancy declined over time^26^. Furthermore, the relationship between conspiracy beliefs and preventive behaviors is not necessarily constant across the pandemic, as it may be influenced by changing regulations (e.g., lockdowns or masking mandates), varying willingness to follow them, and varying mortality rates. Thus, the assumption of stationarity^9^ is probably violated in the present meta-analysis. However, it has been shown that, even under nonstationarity, CoTiMA leads to less biased estimates than other approaches^9^.

Because different instruments were used at different waves in many of the included studies, it was not possible to examine moderators such as type of conspiracy beliefs or type of preventive behavior in the present meta-analysis. Furthermore, researchers often use a combined index of preventive behaviors rather than assessing social distancing, mask wearing and other preventive measures separately. Therefore, it was not possible to make meaningful comparisons between different types of preventive behavior. Nonetheless, examining such differences may be worth pursuing. Bierwiaczonek et al.^8^ reported a stronger negative relationship between conspiracy beliefs and vaccination than between conspiracy beliefs and other types of preventive behavior. One could also expect differences between measures of conspiracy beliefs. Due to content similarity, COVID-19-related conspiracy beliefs could be more strongly associated with COVID-19-related preventive behavior than general conspiracy beliefs. However, Bierwiaczonek and colleagues found mostly miniscule differences between different types of conspiracy beliefs. Although examining static moderators such as measure of preventive behavior or measure of conspiracy beliefs would be straightforward within the CoTiMA framework if more researchers used the same instruments over the course of their study, accounting for time-varying moderators such as changing regulations would be more challenging. Further research, including simulation studies, is needed to offer well-founded recommendations for CoTiMA extensions. Specifically, scenarios including time-varying moderators or allowing multiple operationalizations per study or other dependent effect sizes need to be examined. To investigate the relevance of time-varying moderators, one could also use a continuous time modeling approach based on raw data from primary studies rather than summary statistics. Alternatively, to examine the heterogeneity both between and within studies, one could conduct a standard individual participant data meta-analysis^30^ or an integrative data analysis^31^. Such approaches enable one to conduct analyses that are not restricted to study-level moderators such as mean age of the sample or mean trust value in the sample, as they can utilize information available at the participant level, e.g., trust of person 1, trust of person 2, etc.

According to the meta-analytic evidence, the largest cross-lagged effects can be expected for time lags between 3 and 6 months. However, the “optimal” time lag should be regarded as the most plausible estimate, given the available data. It has been emphasized that differences between the trajectories of individual studies do not solely reflect true heterogeneity but also statistical artifacts. In general, short studies do not yield reliable estimates of cross-lagged effects for long time intervals. Thus, meta-analytic estimates can be biased by the study duration. Although there were several studies that ended after several months rather than several weeks^20,28^, it is possible that a different “optimal” time lag would result had there been even longer primary studies available. Nonetheless, the present meta-analysis enables one to differentiate between good and bad time lags at least on a scale ranging from several weeks to several months.

When interpreting the present findings, it is also important to note that lack of adherence to measures imposed by governments does not necessarily mean that people do not show any preventive behavior. Sometimes, a lack of adherence means that there are no mandated substantive measures, and people find it inadequate^4^. Thus, the measurement of preventive behavior is contaminated by other factors to a certain extent. Furthermore, both preventive behavior and conspiracy beliefs are usually assessed via self-reports, which can introduce some bias. After all, introspection is not always reliable^32^. Nevertheless, other information sources such as internet activity are seldom used when measuring conspiracy beliefs^33^. The research field could benefit from using other information sources, such as spouses or colleagues.

Another aspect that needs to be considered when interpreting the present results pertains to the causal interpretation of the identified patterns. The present findings are based on primary studies with two or more measurement waves, which increases the plausibility of causal claims by ensuring the temporal precedence of potential causes. Nevertheless, the plausibility of causal claims also depends on other factors. Psychological mechanisms are often rather complex, which necessitates considering intermediary variables and multiple causes. Establishing the causal effects of certain variables (e.g., conspiracy beliefs) might require adjusting for specific variables^34^. Interestingly, in some primary studies examining conspiracy beliefs and COVID-19-related preventive behaviors, other potentially relevant variables have been acknowledged, including trust in science and health professionals^23,27^. The reduced willingness to show preventive behaviors was also linked to boldness, dark personality, and perceived barriers to preventive behaviors^2,3^. Furthermore, according to meta-analytic findings^35^, conspiracy beliefs are related to narcissism, paranoia, schizotypy, pseudoscientific beliefs, and religiosity. Thus, accounting for those variables could be fruitful when examining the relationship between conspiracy beliefs and COVID-19-related preventive behaviors. While it is generally possible to examine more complex meta-analytic models using structural equation modeling^36,37^, it requires multiple studies examining similar additional variables. Therefore, such meta-analytic models could not be tested in the present study. Nevertheless, the present findings offer some useful guidance with respect to the magnitude of the cross-lagged effects that one could expect. Furthermore, the current study contributes to the literature by demonstrating the importance of the length of the time interval, which could inform future interventions and studies.

## Data Availability

The datasets generated and/or analyzed during the current study are available in the osf repository, https://osf.io/k4ba9/. *Registration* This meta-analysis was not pre-registered and there is no pre-registration protocol.
